# Dietary dairy product intake and incident type 2 diabetes: a prospective study using dietary data from a 7-day food diary

**DOI:** 10.1007/s00125-014-3176-1

**Published:** 2014-02-08

**Authors:** Laura M. O’Connor, Marleen A. H. Lentjes, Robert N. Luben, Kay-Tee Khaw, Nicholas J. Wareham, Nita G. Forouhi

**Affiliations:** 1 MRC Epidemiology Unit, University of Cambridge School of Clinical Medicine, Institute of Metabolic Science, Cambridge Biomedical Campus, Box 285, Cambridge, CB2 0QQ UK; 2 Department of Public Health and Primary Care, University of Cambridge, Cambridge, UK

**Keywords:** Cheese, Dairy, Fermented dairy, Milk, Type 2 diabetes, Yoghurt

## Abstract

**Aim/hypothesis:**

The aim of this study was to investigate the association between total and types of dairy product intake and risk of developing incident type 2 diabetes, using a food diary.

**Methods:**

A nested case-cohort within the EPIC-Norfolk Study was examined, including a random subcohort (*n* = 4,000) and cases of incident diabetes (*n* = 892, including 143 cases in the subcohort) followed-up for 11 years. Diet was assessed using a prospective 7-day food diary. Total dairy intake (g/day) was estimated and categorised into high-fat (≥3.9%) and low-fat (<3.9% fat) dairy, and by subtype into yoghurt, cheese and milk. Combined fermented dairy product intake (yoghurt, cheese, sour cream) was estimated and categorised into high- and low-fat. Prentice-weighted Cox regression HRs were calculated.

**Results:**

Total dairy, high-fat dairy, milk, cheese and high-fat fermented dairy product intakes were not associated with the development of incident diabetes. Low-fat dairy intake was inversely associated with diabetes in age- and sex-adjusted analyses (tertile [T] 3 vs T1, HR 0.81 [95% CI 0.66, 0.98]), but further adjustment for anthropometric, dietary and diabetes risk factors attenuated this association. In addition, an inverse association was found between diabetes and low-fat fermented dairy product intake (T3 vs T1, HR 0.76 [95% CI 0.60, 0.99]; *p*_trend_ = 0.049) and specifically with yoghurt intake (HR 0.72 [95% CI 0.55, 0.95]; *p*_trend_ = 0.017) in multivariable adjusted analyses.

**Conclusions/interpretation:**

Greater low-fat fermented dairy product intake, largely driven by yoghurt intake, was associated with a decreased risk of type 2 diabetes development in prospective analyses. These findings suggest that the consumption of specific dairy types may be beneficial for the prevention of diabetes, highlighting the importance of food group subtypes for public health messages.

**Electronic supplementary material:**

The online version of this article (doi:10.1007/s00125-014-3176-1) contains peer-reviewed but unedited supplementary material, which is available to authorised users.

## Introduction

Dairy products are important sources of high-quality protein, vitamins (A, D [in fortified products], B_12_, phylloquinones and menaquinones, and riboflavin) and minerals (calcium, magnesium and potassium). However, dairy products are also a source of saturated fats, the intake of which is discouraged in current dietary guidelines, and it is recommended that saturated fat intake be replaced with unsaturated fat intake [1]. Dairy product intake has been associated with a lower risk of type 2 diabetes in three meta-analyses of large prospective epidemiological studies [2–4]. Furthermore, using objective measures, evidence is emerging that dairy derived fatty acids may have a protective effect on the risk of diabetes [5, 6].

Specific dairy subtypes and the intake of low- and high-fat dairy products have been investigated for independent risks with incident diabetes [7–11]. We previously reported that fermented dairy product intake was associated with an inverse risk of diabetes in a study of eight European countries in the European Prospective Investigation of Cancer (EPIC)-Interact study [7]. Furthermore, cheese and fermented dairy products were shown to have an inverse association with glucose regulation measures but not with incident diabetes risk in a study of Danish adults [9]. However, other studies have found no association of dairy subtypes with type 2 diabetes [8, 10, 11]. None of the recent studies that specifically examined low- and high-fat dairy reported an association [8, 9, 11]. Nonetheless, two meta-analyses showed an inverse association of low-fat dairy and incident diabetes but a null association of high-fat dairy intake and disease development [2, 4]. In addition, a recent review concluded that there is inconsistent evidence to suggest that high-fat dairy product consumption was associated with increased risk of incident diabetes [12]. Thus, the nature of the association between dairy product intake and type 2 diabetes remains unclear.

To date, research in this area has made use of retrospective food frequency questionnaires (FFQs) to estimate dairy intake. Allowing for standardised responses, FFQs offer a pragmatic choice as they enable dietary data in large studies to be collected relatively inexpensively, and are useful in examining diet–disease relationships as individuals are ranked according to intake. However, FFQs are limited by the predefined food lists when analysing type and variety of foods. In addition, their retrospective nature involves a recall period, often of 12 months, which poses problems of recall bias and related misreporting error. By contrast, food diaries are not restricted in the types and amounts of foods that can be recorded at the time of consumption, reducing recall bias and providing extra detail which, along with their prospective nature may help to better investigate the association between risk of diabetes development and total dairy, low- and high-fat dairy and dairy subtypes. Currently, no studies on dairy product intake and type 2 diabetes have made use of food diaries.

The primary aim of this work was to examine the associations of types and amount of dairy product intake with incident type 2 diabetes using 7 day food diary data. A secondary aim was to examine the effect of substituting a portion of dairy for an alternative food on risk of incident type 2 diabetes.

## Methods

### Study design and population

The EPIC-Norfolk study, described in detail previously [13], is a UK population-based cohort of 25,639 men and women aged 40–79 years at baseline, recruited in 1993–1997. All volunteers gave written informed consent, and the study was approved by the Norfolk Research Ethics Committee. Participants attended a baseline health examination at their general practitioner’s clinic, after which follow-up data collection points included a postal questionnaire at 18 months, a second health examination visit in 1998–2000 and a postal questionnaire in 2002–2004.

A nested case-cohort was designed, including 4,000 subcohort participants selected at random from the entire cohort, and 892 incident diabetes cases were ascertained. Due to the randomly selected nature of the subcohort, 143 of these cases were included within the subcohort, which the case-cohort design allows and accounts for in the analysis.

For the current analyses we excluded those with prevalent and uncertain diabetes status (*n* = 83), those with missing food diary data (*n* = 18) and other covariates (*n* = 3), and those with an implausible ratio of energy intake to basal metabolic rate as defined using published equations [14] (*n* = 82; top and bottom 1% of the distribution). Individuals with prevalent myocardial infarction, stroke and cancer were also excluded (*n* = 436) to account for possible post-diagnosis changes in diet. Therefore, a total of 4,127 participants (753 cases and 3,502 subcohort, including 128 subcohort cases) remained for analysis.

### Case ascertainment and verification

Incident type 2 diabetes cases until 31 July 2006 were ascertained using multiple data sources, including self-report of doctor-diagnosed diabetes from the second health check or follow-up health and lifestyle questionnaires, self-report of diabetes-specific medication in either of the two follow-up questionnaires or medication brought to the follow-up health check. These were verified through record linkage with the general practice diabetes register, local hospital diabetes register, hospital admissions data and Office of National Statistics mortality data with coding for diabetes. Participants who self-reported a history of diabetes that could not be verified with any other sources of ascertainment were not included as confirmed cases of diabetes.

### Dietary intake

Baseline dietary intake data were collected using a 7 day food diary [15]. Food weights were estimated using photographs representing portion sizes, household measures and standard units. Nurses trained to standardised protocols provided participants with instructions on how to complete the diary at the health check and asked participants to recall the previous day’s intake. This formed day one of the diary. Participants prospectively completed the remaining 6 days and sent it back to the study centre. Food intake data were entered using the Data into Nutrients for Epidemiologic Research (DINER) entry system [16] and converted into food weights and nutrient intakes by DINERMO [17].

A pragmatic approach was applied in estimating dairy product intake. Total dairy intake was estimated as food items that only consist of dairy plus composite dishes where dairy was the main ingredient. Ice cream, chocolate, butter used in cooking and dairy included as a minor ingredient in composite dishes were not included. Intakes were categorised into high- and low-fat dairy using 3.9% fat (the fat content of whole milk in the UK) as a cut-off point. Intakes were also categorised by subtype into yoghurt, cheese and milk intakes. A non-exclusive group, total fermented dairy products, was created and subdivided into high- and low-fat fermented dairy products using 3.9% fat as a cut-off point. Category descriptions are detailed in Table 1.

**Table 1:** Dairy intake classification: EPIC-Norfolk study

Total dairy^a^	Butter, cheese, cream, crème fraîche, dried/powdered milk (made up weight), evaporated milk, milk, sour cream, yoghurt, baby milk
High-fat dairy (≥3.9% fat)	Butter, full fat unripened cheese, all hard, processed and soft cheese, cream, sour cream, crème fraîche, whole milk, whole dried/powdered milk (made up weight), all evaporated milk, baby milk
Low-fat dairy (<3.9% fat)	Low-fat unripened cheese, semi-skimmed milk, skimmed milk, dried semi-skimmed/skimmed milk, all yoghurt
Milk	Liquid and powdered/dried milk (made up weight); cow, sheep and goat sources
Cheese	Hard, processed, soft (e.g. brie) and unripened (e.g. mozzarella, fromage frais, ricotta)
Yoghurt	Full-, low-, reduced-, 0%- fat varieties
Total fermented dairy products	All yoghurt, all cheese, sour cream and crème fraîche
High-fat fermented dairy products (≥3.9% fat)	Hard cheese, soft cheese, high-fat unripened cheese, sour cream, crème fraîche
Low-fat fermented dairy products (<3.9% fat)	All yoghurt, low-fat unripened cheeses (e.g. fromage frais, low-fat cottage cheese)

^a^Estimated as food items that only consist of dairy plus composite dishes where dairy was the main ingredient

### Covariates

Baseline demographic, lifestyle and health characteristics were collected using a self-administered questionnaire. A validated four-point physical activity index was used to categorise participants as active, moderately active, moderately inactive or inactive [18]. Height, weight, waist circumference and systolic and diastolic BP were measured, BMI was calculated and blood samples were collected using standardised procedures. The questionnaires, physical activity index and anthropometric measurements methods have been previously described in detail [13]. Dietary covariates were estimated using data from the 7-day food diary. Plasma vitamin C measurement is a marker of recent fruit and vegetable intake [19] and provides an indication of dietary quality. To determine plasma vitamin C levels, venous blood was drawn from non-fasting participants into citrate tubes and stored overnight in a dark container at 4–7°C. Samples were centrifuged and plasma was stabilised using a standardised volume of metaphosphoric acid and measured using a fluorometric assay.

### Statistical analysis

Dairy product intakes were divided into tertiles according to the subcohort intake distribution. Baseline characteristics and dietary intakes were examined across tertiles of total dairy intake in the subcohort. Dairy product intake was adjusted for energy intake using the residual method [20]. The residuals from the regression of dairy intake on total energy intake were rescaled by adding the expected dairy intake for a person with mean total energy intake. By design, 128 incident diabetes cases were included in the random subcohort. To account for this case-cohort design, Prentice-weighted Cox regression models [21, 22] were used to calculate HRs and 95% CIs for the association between dairy intake and incident type 2 diabetes. Age was included as the underlying timescale in the Cox models, with entry time defined as age at recruitment and exit time as age at diagnosis of diabetes, death, loss to follow-up or censoring at the end of follow-up, whichever came first.

Model 1 adjusted for age (continuous, as underlying timescale) and sex. Model 2 additionally adjusted for BMI (continuous), family history of diabetes (yes or no), smoking status (current, former, never), usual alcohol consumption (continuous units/week) estimated from a health questionnaire, physical activity index (inactive, moderately inactive, moderately active, active), social class (professional, managerial, skilled, semi-skilled, unskilled) and education level (no qualification, O level, A level, degree or higher). Model 3 additionally adjusted for dietary covariates, including energy intake (continuous kJ/day), and intake of fibre, fruit, vegetables, red meat, processed meat and coffee (all continuous g/day). To test for linearity the median intake value of each tertile of dairy intake was included in the Cox regression model. The assumption of proportional hazards, checked by including time-dependent covariates in the model, was not violated. Possible interactions with sex, BMI, physical activity index and smoking status were examined by including the interaction terms in the most adjusted models.

The independence of the associations of specific dairy subtypes was tested by mutually adjusting for other dairy subtypes and separately for all other food groups. In a further model, we included hypertension (dichotomous >140 mmHg systolic BP or >90 mmHg diastolic BP, or on hypertension medication), hypercholesterolaemia (dichotomous >6.2 mmol/l or on lipid-lowering medication), sugar-sweetened beverage intake and *trans* fat intake. The potential mediating roles of saturated fat, vitamin D, calcium and magnesium in the association of dairy intake and type 2 diabetes were examined by entering these into further models. To test the effect of substituting dairy products for alternative foods, we set a priori criteria of including only those dairy products that were associated with type 2 diabetes and considering foods that would be likely replacements. The effect of substituting a portion of one food for another was examined by including both as continuous variables in a multivariable model (model 2 plus energy [kJ]). The difference in their beta coefficients and their variances and covariance were used to estimate the beta coefficient and variance for the substitution effect, which in turn was used to calculate HRs and 95% CIs [23]. Portion sizes are means of those used in DINERMO [17].

Sensitivity analyses included repeating the models without the residual method for energy adjustment (i.e. using absolute intakes), restricting analyses to dairy product consumers only and including plasma vitamin C levels. In addition, the analyses were repeated excluding participants diagnosed with incident diabetes in the first 2 years of follow-up in order to minimise the possibility of reverse causality and those classified as energy misreporters according to published cut-offs for the ratio of energy intake to basal metabolic rate [24], and including those with prevalent chronic diseases at baseline. Participants with high dairy product intakes were not excluded as the intakes were deemed plausible and, when examined in consumers only, no intakes were more than 1 SD higher than the median.

The analyses were performed using Stata (version 12; Stata Corp, College Station, TX, USA).

## Results

The largest contributors to dairy intake (g) were milk (81.7%), cheese (8.7%) and yoghurt (7.6%). The mean (SD) estimated total dairy intake was 269 g/day (160 g/day) in the subcohort, and 65% of total dairy intake was from low-fat sources. Those with higher estimated dairy product intakes were more likely to be men, have lower BMI and waist circumference (men only), drink less alcohol, smoke less, be more physically active, have higher education levels, and have higher intakes of energy, calcium, magnesium, vitamin D, fibre, fruit and vegetables and lower intakes of monounsaturated and polyunsaturated fat (percentage total energy) and meat (red and processed; Table 2). Patterns of baseline characteristics were similar (to those across tertiles of total dairy intake) when examined across tertiles of milk intake and for the most part across tertiles of yoghurt intake (electronic supplementary material [ESM] Table 1). Those with higher amounts of yoghurt consumption were more likely to be women and have lower intakes of saturated fat (percentage total energy), and there was no variation with BMI or waist circumference. Baseline characteristics by tertile of cheese intake showed no difference in BMI or waist circumference with increasing intake of cheese, whereas smoking prevalence and monounsaturated and polyunsaturated fat intake increased, and these participants were more likely to consume more alcohol. Dairy product intakes were not correlated with each other (Spearman’s *ρ* all <0.08).

**Table 2:** Baseline characteristics in the subcohort (*n* = 3,502) by tertile of total dairy intake: EPIC-Norfolk study

Variable	Total dairy intake (g/day)^a^			
	Tertile 1 (*n* = 1,168)	Tertile 2 (*n* = 1,167)	Tertile 3 (*n* = 1,167)	
	≤183	184–312	≥313	*p* value^b^
Total dairy intake (g/day)^c^	116 ± 45	245 ± 38	447 ± 134	
*Anthropometric and socio-demographic characteristics*				
Men^d^	473 (40.5)	480 (41.1)	566 (48.5)	<0.001
Age (years)^c^	59 ± 9	59 ± 10	59 ± 9	0.902
Waist circumference (cm)				
Men^c^	97.5 ± 10.5	95.7 ± 9.4	93.9 ± 8.7	<0.001
Women^c^	82.4 ± 11.4	81.4 ± 10.49	81.3 ± 9.8	0.125
BMI (kg/m^2^)^c^	26.8 ± 4.2	26.2 ± 3.8	25.8 ± 3.5	<0.001
Alcohol (units/week)^e^	8.5 (1.0, 12.0)	6.6 (1, 9.5)	5.4 (1.0, 7.5)	<0.001
Smoking: current^d^	163 (14.1)	124 (10.7)	111 (9.6)	<0.001
Education: degree or higher^d^	156 (13.4)	157 (13.5)	164 (14.1)	0.047
Physical activity: active^d^	182 (15.6)	232 (19.9)	254 (21.8)	<0.001
Social class: professional/managerial^d^	495 (42.4)	509 (43.6)	490 (42.0)	0.709
*Dietary intake*				
Energy (kJ/day)^c^	7,519 ± 2,092	8,171 ± 2,021	8,912 ± 2,230	<0.001
Saturated fat (% TE)^c^	12.9 ± 3.1	12.9 ± 3.0	13.1 ± 3.1	0.145
Monounsaturated fat (% TE)^c^	12.3 ± 2.4	11.9 ± 2.1	11.6 ± 2.0	<0.001
Polyunsaturated fat (% TE)^c^	6.7 ± 1.8	6.6 ± 1.7	6.2 ± 1.7	<0.001
Calcium (mg/day)^c^	624 ± 186	807 ± 175	1,077 ± 256	<0.001
Magnesium (mg/day)^c^	256 ± 75	289 ± 78	330 ± 87	<0.001
Vitamin D (μg/day)^e^	2.38 (1.53, 3.67)	2.85 (1.83, 4.30)	2.98 (2.07, 4.50)	<0.001
Fibre (g/day)^c^	13.46 ± 4.72	15.04 ± 4.95	16.47 ± 5.90	<0.001
Fruit (g/day)^c^	141 ± 124	165 ± 131	173 ± 130	<0.001
Vegetables (g/day)^e^	74 (44, 119)	82 (48, 124)	83 (50, 126)	0.007
Processed meat (g/day)^c^	30 ± 28	28 ± 24	26 ± 26	0.010
Red meat (g/day)^e^	11 (0, 30)	13 (0, 25)	9 (0, 23)	0.008
Coffee (g/day)^e^	304 (80, 598)	301 (64, 477)	271 (46, 582)	0.342
Plasma vitamin C level (μmol/l)^c^	53 ± 20	54 ± 20	54 ± 20	0.357

^a^Tertile cut-offs are based on absolute intakes

^b^ *p value* as calculated using ANOVA or Kruskal–Wallis ANOVA for non-parametric data

^c^Mean ± SD; all values in row

^d^ *n* (%); all values in row

^e^Median (interquartile range); all values in row

TE, total energy

Total dairy, high-fat dairy, milk (whole milk or reduced-fat milk, data not shown), cheese or high-fat fermented dairy product intakes (energy-adjusted g/day) were not associated with the hazard of type 2 diabetes (Table 3). Low-fat dairy intake was inversely associated with the hazard of type 2 diabetes in model 1 (*p*_trend_ = 0.025) with an HR of 0.81 (95% CI 0.66, 0.98) comparing the highest with the lowest tertile of intake. Adjustment for confounders attenuated the association to null (model 3 HR 0.92 [95% CI 0.73, 1.17]). Total fermented dairy product intake was associated with 19% lower hazard of type 2 diabetes in the age- and sex-adjusted model. This became non-significant with further adjustment for potential confounders. When only low-fat fermented dairy product intake was examined there was a significant inverse association in adjusted analysis: a comparison of the highest tertile with the lowest tertile in the most adjusted model gave an HR of 0.76 (95% CI 0.60, 0.99; *p*_trend_ = 0.049). Yoghurt intake was inversely associated with the hazard of diabetes when adjusted for age and sex (tertile 3 vs tertile 1; HR 0.65 [95% CI 0.52, 0.83]; *p*_trend_ < 0.001). This lower hazard of diabetes was maintained through further adjustment for potential confounders (model 3 HR 0.72 [95% CI 0.55, 0.95]; *p*_trend_ = 0.017).

**Table 3:** HRs for the association of dairy product intake and risk of type 2 diabetes per category of energy-adjusted dairy product intake using the residual method: EPIC-Norfolk Study (*n* = 4,127)

	Tertile 1^a^	Tertile 2	Tertile 3	*p*_*trend*_
Total dairy intake (g/day)^b^	130 (0–190)	246 (190–311)	404 (311–1,544)	
Cases/*n*	278/1,390	240/1,374	234/1,362	
Model 1	1	0.89 (0.74,1.08)	0.85 (0.70,1.04)	0.100
Model 2	1	0.93 (0.74,1.18)	1.04 (0.83,1.31)	0.732
Model 3	1	0.94 (0.75,1.19)	1.08 (0.86,1.37)	0.537
High-fat dairy intake (g/day)^b^	0 (0–19)	35 (19–54)	125 (54–1,095)	
Cases/*n*	257/1,377	205/1,334	291/1,416	
Model 1	1	0.90 (0.73,1.11)	1.15 (0.95,1.39)	0.054
Model 2	1	0.76 (0.60,0.98)	1.11 (0.89,1.38)	0.075
Model 3	1	0.76 (0.58,0.98)	1.09 (0.87,1.37)	0.080
Low-fat dairy intake (g/day)^b^	16 (0–89)	160 (89–230)	325 (230–1,424)	
Cases/*n*	286/1,404	253/1,379	214/1,344	
Model 1	1	0.97 (0.80,1.17)	0.81 (0.66,0.98)	0.025
Model 2	1	0.92 (0.74,1.16)	0.89 (0.71,1.11)	0.305
Model 3	1	0.95 (0.76,1.20)	0.92 (0.73,1.17)	0.540
Milk intake (g/day)^b^	97 (0–150)	201 (150–264)	353 (264–1,518)	
Cases/*n*	271/1,387	242/1,375	238/1,363	
Model 1	1	0.89 (0.73,1.08)	0.86 (0.71,1.04)	0.104
Model 2	1	0.94 (0.75,1.18)	1.08 (0.86,1.36)	0.551
Model 3	1	0.95 (0.76,1.20)	1.11 (0.88,1.41)	0.432
Yoghurt intake (g/day)^b^	0 (0)	21.5 (0.1–44)	80 (44–513)	
Cases/*n*	542/2,698	117/723	94/706	
Model 1	1	0.79 (0.63,0.99)	0.65 (0.52,0.83)	<0.001
Model 2	1	0.83 (0.63,1.08)	0.70 (0.54,0.91)	0.007
Model 3	1	0.84 (0.64,1.10)	0.72 (0.55,0.95)	0.017
Cheese intake (g/day)^b^	3 (0–8)	13 (8–20)	32 (20–364)	
Cases/*n*	247/1,371	249/1,366	257/1,390	
Model 1	1	0.98 (0.81,1.20)	1.04 (0.86,1.27)	0.623
Model 2	1	1.05 (0.84,1.31)	1.02 (0.81,1.28)	0.946
Model 3	1	1.05 (0.84,1.32)	1.04 (0.83,1.31)	0.760
Total fermented dairy intake (g/day)^b^	4 (0–13)	23 (13–40)	76 (40–518)	
Cases/*n*	269/1,388	277/1,397	207/1,342	
Model 1	1	1.01 (0.84,1.22)	0.81 (0.66,0.98)	0.018
Model 2	1	1.02 (0.82,1.27)	0.82 (0.65,1.04)	0.064
Model 3	1	1.04 (0.84,1.29)	0.85 (0.68,1.08)	0.120
High-fat fermented dairy intake (g/day)^b^	0 (0)	8 (0.1, 14)	22 (14-185)	
Cases/*n*	194/1,064	283/1,527	276/1,536	
Model 1	1	0.96 (0.78,1.18)	0.96 (0.79,1.18)	0.790
Model 2	1	1.19 (0.94,1.51)	1.12 (0.88,1.43)	0.514
Model 3	1	1.23 (0.96,1.57)	1.16 (0.91,1.49)	0.397
Low-fat fermented dairy intake (g/day)^b^	0 (0)	21 (0.1–43)	80 (43–513)	
Cases/*n*	510/2,538	130/800	113/789	
Model 1	1	0.81 (0.66,1.01)	0.72 (0.58,0.90)	<0.001
Model 2	1	0.87 (0.67,1.13)	0.74 (0.58,0.96)	0.020
Model 3	1	0.89 (0.69,1.16)	0.76 (0.60,0.99)	0.049

Values are HR (95% CI) except where indicated otherwise

Model 1: adjusted for age (as underlying timescale), sex

Model 2: Model 1 plus BMI, family history of diabetes, smoking, alcohol, physical activity index, social class, education level

Model 3: Model 2 plus energy, fibre, fruit, vegetables, red meat, processed meat, coffee intake

^a^Tertile cut-offs are based on energy-adjusted intakes in the subcohort, calculated using the residual method

^b^Median intake (range); all values in row

No interactions of total dairy or dairy subtypes with sex, BMI, physical activity index or smoking status were evident. Results were similar for total dairy and dairy subtypes when BMI was replaced with waist circumference. The independence of the associations of dairy subtypes with diabetes was not affected by adjustment for other dairy subtypes in the multivariable model. Mutual adjustment for all other food groups had no effect on the association of low-fat fermented dairy or yoghurt intake (tertile 3 vs tertile 1). Similarly, further adjustment of the multivariable model for hypertension, hypercholesterolaemia, sugar-sweetened beverage intake and *trans* fat intake did not materially change the results (not shown). The addition of saturated fat (percentage total energy) to the multivariable model marginally attenuated the associations for intakes of low-fat fermented dairy products (model 3 HR 0.78 [95% CI 0.61, 1.01]; *p*_trend_ = 0.058) and yoghurt (model 3 HR 0.73 [95% CI 0.56, 0.95]; *p*_trend_ = 0.020). When vitamin D, calcium and magnesium were entered together into a further model the association of total dairy and all dairy subtypes with type 2 diabetes became marginally more inverse, but there was no change in the significance of the associations (results not shown).

Table 4 shows the HRs (95% CI) for substituting one portion of yoghurt for one portion of an alternative dessert or snack (described in ESM Table 2). Substituting yoghurt for snacks was associated with 47% lower hazard of type 2 diabetes. No alternative dessert substitution was significant.

**Table 4:** HRs for risk of type 2 diabetes associated with the substitution of yoghurt (137 g^a^) for snacks and desserts: EPIC-Norfolk study (*n* = 4,127)

Dairy desserts (146 g)	0.69	(0.32, 1.06)
Puddings (138 g)	0.87	(0.21, 1.53)
Cake (76 g)	0.91	(0.49, 1.32)
Biscuits (17 g)	0.77	(0.44, 1.09)
Snacks (32 g)	0.53	(0.23, 0.84)
Creams (23 g)	0.95	(0.40, 1.48)
Chocolate confectionary (25 g)	0.71	(0.39, 1.03)
Other desserts (113 g)	0.83	(0.04, 1.63)

Values are given as HR (95% CI)

HRs are adjusted for age (as underlying timescale), sex, BMI, family history of diabetes, physical activity index, social class, education level and energy

^a^Mean portion sizes as used in DINERMO [17]

### Sensitivity analyses

When the analyses were repeated including dairy product consumers only (for each dairy subtype), associations with incident diabetes became non-significant but remained in the same direction as for the total sample. Repeating the analyses using absolute intakes of dairy products, rather than the energy-adjusted dairy intake, as the main exposure variables did not materially change the associations, nor did including plasma vitamin C as an objective marker of dietary quality, excluding those diagnosed with type 2 diabetes within the first 2 years of follow-up or excluding misreporters of energy intake. When participants with prevalent chronic disease (*n* = 436) were included, the associations were attenuated and only the association with yoghurt intake remained significant (tertile 3 vs tertile 1, model 3 HR 0.72 [95% CI 0.55, 0.95]).

## Discussion

### Current findings in context

In this prospective study using dietary intake data from 7-day food diaries, being in the highest tertile of low-fat fermented dairy product intake (equivalent to consuming median 80 g/day) was associated with a 24% decreased risk of developing incident diabetes compared with being a non-consumer. In public health terms this equates to 4.5 standard size portions (125 g) per week of low-fat fermented dairy products, largely comprising of yoghurt (all types) and including low-fat unripened cheese such as low-fat cottage cheese and fromage frais. This finding was independent of age, sex, family history of diabetes, BMI, lifestyle factors and other dietary intakes associated with diabetes. Total dairy, high-fat dairy, milk, cheese and high-fat fermented dairy product intakes were not associated with incident diabetes in these analyses.

Largely owing to its saturated fat content, dairy intake is conventionally perceived as having an adverse impact on health outcomes; for instance it is scored as an ‘unhealthy’ item in the Mediterranean diet pattern score [25]. However, dairy products contain many nutrients including calcium, vitamin D [26] and magnesium [27] that are considered healthy constituents of dairy products. Similar to our previously published work using EPIC-InterAct data [7], the inclusion of these nutrients in our current analyses strengthened the associations with a lower hazard of type 2 diabetes for all dairy subtypes, suggesting the presence of other favourable dairy components. In the current analysis the inclusion of saturated fat in the most adjusted model marginally attenuated the hazard of type 2 diabetes, nonetheless associations remained significant.

Healthy and unhealthy dietary intake habits are known to cluster, but in this analysis mutually adjusting for other food groups had no effect on associations. We also examined the effect of choosing yoghurt (as the only homogenous food group associated with diabetes in this analysis) instead of an alternative dessert or snack, to examine the possibility that the reduction in risk does not come from yoghurt consumption but from the absence of the alternative food. Replacing a portion of snacks (crisps) with a portion of yoghurt reduced the hazard of type 2 diabetes by 47%, suggesting that some of the association may be attributed to not consuming unhealthy alternatives.

Intake of total dairy has been reported to be associated with decreased risk of type 2 diabetes in three meta-analyses [2–4], but no such association was found in the current study. The association with some but not all dairy subtypes may explain this null effect. Findings of recent studies that have focused on specific dairy subtypes and risk of diabetes are not in accord. The Whitehall study of London-based men and women in the civil service showed no relationship between any dairy subtype and incident diabetes [8], nor did a prospective study of Australian adults [10]. Previously, we reported an inverse association between diabetes and intake of fermented dairy products, and a marginal inverse association with cheese intake in the EPIC-InterAct study [7]. Similarly, a study of Danish adults reported an inverse association of fermented dairy intake with fasting plasma glucose and HbA_1c_ levels, as well as cheese intake with incident diabetes and 2 h plasma glucose levels [9]. In the current study, we report null associations for total fermented dairy and cheese intakes, but an inverse association for low-fat fermented dairy intake, which includes unripened cheese, suggesting that previous findings from FFQ data may have been driven by low-fat fermented dairy intakes. Heterogeneity within dairy subtypes between populations may explain the differences in findings. The association of low-fat fermented dairy in the current study is thought to largely be driven by yoghurt intake, as yoghurt accounted for 87% of low-fat fermented dairy consumed (g) and, when examined separately, was associated with a 28% reduced risk of incident diabetes.

Results of the meta-analysis by Tong et al showed low-fat dairy intake was associated with a larger magnitude of a decreased risk in type 2 diabetes (combined RR: 0.82 [95% CI 0.74, 0.90] in a comparison of the highest and lowest intake categories) compared with total dairy (combined RR 0.86 [95% CI 0.79, 0.92]) [2]. Similar associations were shown in the meta-analysis by Aune et al in their dose response analysis (per 400 g total dairy products combined RR 0.93 [95% CI 0.87, 0.99] and per 200 g low-fat dairy products combined RR 0.91 [95% CI 0.86, 0.96]) [4]. Conversely, higher intakes of low-fat dairy were associated with higher risks of chronic disease, in particular type 2 diabetes, in a large prospective study of German adults [28], while regular-fat dairy was associated with a reduced risk of incident metabolic syndrome but not diabetes in a study of Australian older adults [11]. In the current study, low-fat dairy intakes were associated with lower risk of type 2 diabetes only in the age- and sex-adjusted analysis and not after further adjustment for anthropometric, dietary and diabetes risk factors. This might be attributable to lower statistical power of the multivariable model, although differences in classification into low- and high-fat dairy may also explain differences in findings to date, particularly as this can be carried out by fat variety (full- and reduced-fat varieties of dairy subtypes) or by absolute fat content.

### Mechanisms

In the current study, only dairy products that have undergone fermentation were associated with reduced risk of diabetes. Several potential mechanisms for such an association exist. Menaquinones (vitamin K_2_) synthesised by animal tissue are present in dairy products and have been associated with reduced risk of type 2 diabetes [29]. Menaquinones can also be of microbiological origin and these are found primarily in fermented foods. Probiotic bacteria, also present in fermented dairy products, have been shown to improve the lipid profile and antioxidant status in individuals with type 2 diabetes [30, 31], and have beneficial effects on cholesterol levels [32]. Moreover, these low-fat fermented dairy products are naturally low in fat and high in water content and are, therefore, low energy-dense foods. Studies have shown an independent association of low energy-dense foods with lower fasting insulin levels and the metabolic syndrome [33] and a lower risk of type 2 diabetes [34].

### Strengths and limitations

To the best of our knowledge this is the first study of dairy intake and type 2 diabetes to make use of prospective food diary intake data, while past research has used retrospective FFQ data. The detailed nature of food diaries allows for differentiation between subtypes of dairy intake, the inclusion of composite dishes in dairy intake estimates and the identification of probable food choice alternatives for inclusion in substitution analyses. Other strengths include the large study size from which the cohort was drawn and the medical verification of diabetes status using external sources, which meant that loss to follow-up within the study did not affect the completion of case ascertainment.

Limitations of this study warrant consideration. As dietary intake was self-reported and collected prospectively for 7 days the possibility of misreporting food intake and portion sizes and/or changing usual dietary behaviours exists, in particular through participant burden due to the detailed nature of food diaries. However, accounting for energy misreporters in this analysis had little effect on the associations found. We only assessed diet once at baseline thus our estimates do not account for any dietary modifications over the follow-up period. Dairy products consumed as minor ingredients in composite dishes are not captured. Where fat content of individual products or specific brands differed substantially from the average composition of the dairy product, misclassification into low- and high-fat dairy categories may have occurred, however the number of such products is likely to be low. Residual confounding and clustering of healthy behaviours cannot be totally accounted for although the inclusion of a range of covariates in the study allowed for adjustment for anthropometric, lifestyle, other diabetes risk factors and dietary factors including measured plasma vitamin C level. Furthermore, as the study population is predominantly white European in origin the findings cannot be generalised to other populations.

### Conclusions

In this large prospective study, greater low-fat fermented dairy product intake, largely driven by yoghurt intake, was associated with a decreased risk of type 2 diabetes. These findings suggest that the consumption of specific dairy types may be beneficial for the prevention of diabetes and have implications, including the importance of considering food group subtypes, for public health messages.

## Electronic supplementary material

Below is the link to the electronic supplementary material.

ESM Table 1 (PDF 20.9 kb)

ESM Table 2 (PDF 11.3 kb)

## Abbreviations

DINER: Data into Nutrients for Epidemiologic Research
EPIC: European Prospective Investigation of Cancer
FFQ: Food Frequency Questionnaire
