# Supplementary material for: Dietary dairy product intake and incident type 2 diabetes: a prospective study using dietary data from a 7-day food diary
Source: Diabetologia. 2014 Feb 8;57(5):909–17. doi: 10.1007/s00125-014-3176-1 (PMC3980034; doi:10.1007/s00125-014-3176-1)
Supplement: Supplementary file 1 — (PDF 20.9 kb) [file 125_2014_3176_MOESM1_ESM.pdf]

ESM Table 1 Baseline characteristics in the subcohort (n=3,502) by tertile of yoghurt intake: EPIC-Norfolk Study

Variable	Yoghurt intake (g/d)[a] Tertile 1 (n=2,243) 0	Tertile 2 (n=632) 0.9-42.8	Tertile 3 (n=627) 43.0-514.3	p-value[b]
Total dairy intake (g/day)[c]	245 ± 160	276 ± 135	347 ± 159	<0.001
Anthropometric and socio-demographic characteristics				
Men[d]	1,089 (48.6)	226 (35.8)	204 (32.5)	<0.001
Age (years)[c]	59 ± 10	59 ± 9	59 ± 9	0.941
Waist circumference (cm)				
Men[c]	95.8 ± 9.9	95.0 ± 9.2	95.0 ± 8.9	0.317
Women[c]	81.8 ± 11.0	81.4 ± 10.4	81.7 ± 9.7	0.793
BMI (kg/m^2)[c]	26.3 ± 3.9	26.1 ± 3.9	26.3 ± 3.6	0.290
Alcohol (units/week)[e]	4.0 (1.0, 10.5)	4.0 (1.5, 9.0)	2.5 (1.0, 7.5)	<0.001
Smoking: current[d]	297 (13.4)	57 (9.06)	44 (7.04)	<0.001
Education: degree or higher[d]	276 (12.3)	106 (16.8)	95 (15.2)	0.002
Physical activity: Active[d]	424 (18.9)	116 (18.4)	128 (20.4)	0.037
Social class: Professional/managerial[d]	936 (41.7)	294 (46.5)	264 (42.1)	0.094
Dietary intakes				
Energy (kJ/day)[c]	8,251 ± 2272	8,171 ± 1979	8,050 ± 2071	0.142
Saturated fat (% TE)[c]	13.3 ± 3.2	12.8 ± 2.6	12.2 ± 2.9	<0.001
Monounsaturated fat (% TE)[c]	12.1 ± 2.2	11.9 ± 2.0	11.2 ± 2.1	<0.001
Polyunsaturated fat (% TE)[c]	6.6 ± 1.8	6.6 ± 1.6	6.2 ± 1.6	<0.001
Calcium (mg/day)[c]	799 ± 285	850 ± 236	952 ± 268	<0.001
Magnesium (mg/day)[c]	283 ± 84	299 ± 80	314 ± 89	<0.001
Vitamin D (µg/day)[e]	2.75 (1.79, 4.23)	2.78 (1.90, 4.17)	2.73 (1.67, 3.95)	0.435
Fibre (g/day)[c]	14.44 ± 5.25	15.62 ± 5.24	16.32 ± 5.57	<0.001
Fruit (g/day)[c]	143 ± 124	179 ± 127	199 ± 139	<0.001
Vegetables (g/day)[e]	79 (46, 120)	81 (49, 123)	88 (54, 139)	<0.001
Processed meat (g/day)[c]	29 ± 28	26 ± 22	25 ± 25	0.012
Red meat (g/day)[e]	12 (0, 27)	8 (0, 24)	9 (0, 22)	0.006
Coffee (g/day)[e]	279 (40, 568)	322 (104, 598)	303 (80, 608)	0.003

[a]Tertile cut-offs are based on absolute intakes of total dairy

[b]*p*-value as calculated using ANOVA or Kruskal-Wallis ANOVA for non-parametric data

[c]Mean ± SD; all such values

[d]n; % in parentheses; all such values

[e]Median; interquartile range in parentheses; all such values

TE, total energy
