# Supplementary material for: Dietary dairy product intake and incident type 2 diabetes: a prospective study using dietary data from a 7-day food diary
Source: Diabetologia. 2014 Feb 8;57(5):909–17. doi: 10.1007/s00125-014-3176-1 (PMC3980034; doi:10.1007/s00125-014-3176-1)
Supplement: Supplementary file 2 — (PDF 11.3 kb) [file 125_2014_3176_MOESM2_ESM.pdf]

ESM Table 2 Description of snacks & dessert food groups: EPIC-Norfolk Study

Cake	Cakes, pies, pastries, scones, muffins, chocolate cakes, éclairs, pastries with chocolate
Dairy desserts	Chocolate and non-chocolate dairy based desserts/sauces, ice-creams
Puddings	Cereal and chocolate based puddings, crumble, sponge pudding, suet pudding
Biscuits	Chocolate and non-chocolate biscuits, with fruit, nuts, filled etc, including cereal bars
Snacks	Crisps and other potato based snacks, bread/pastry type snacks (cheese pastry etc.)
Creams	Dairy creams (double, single, whipping/whipped, half fat, clotted, crème fraîche, dream topping) and non-dairy creams (Delight, Elmlea, Dessert Top, Tip Top soya cream)
Chocolate confectionary	Chocolate bars, chocolate sweets
Other desserts	Ice-lollies, sorbets, jelly, sweet egg dishes (meringues, soufflés)
